# Catalytic Effects of Temperature and Silicon Dioxide Nanoparticles on the Acceleration of Production from Carbonate Rocks

**DOI:** 10.3390/nano11071642

**Published:** 2021-06-23

**Authors:** Ibraheem Salaudeen, Muhammad Rehan Hashmet, Peyman Pourafshary

**Affiliations:** School of Mining and Geosciences, Nazarbayev University, Nur-Sultan 010000, Kazakhstan; ibraheem.salaudeen@nu.edu.kz (I.S.); peyman.pourafshary@nu.edu.kz (P.P.)

**Keywords:** hybrid nanofluid injection, wettability alteration, carbonate reservoirs, engineered water, SiO_2_ nanofluid

## Abstract

The use of engineered water (EW) nanofluid flooding in carbonates is a new enhanced oil recovery (EOR) hybrid technique that has yet to be extensively investigated. In this research, we investigated the combined effects of EW and nanofluid flooding on oil-brine-rock interactions and recovery from carbonate reservoirs at different temperatures. EW was used as dispersant for SiO_2_ nanoparticles (NPs), and a series of characterisation experiments were performed to determine the optimum formulations of EW and NP for injection into the porous media. The EW reduced the contact angle and changed the rock wettability from the oil-wet condition to an intermediate state at ambient temperature. However, in the presence of NPs, the contact angle was reduced further, to very low values. When the effects of temperature were considered, the wettability changed more rapidly from a hydrophobic state to a hydrophilic one. Oil displacement was studied by injection of the optimised EW, followed by an EW-nanofluid mixture. An additional recovery of 20% of the original oil in place was achieved. The temperature effects mean that these mechanisms are catalytic, and the process involves the initiation and activation of multiple mechanisms that are not activated at lower temperatures and in each standalone technique.

## 1. Introduction

Increased global demand for fossil fuels and a continuous decline in reserves have made enhanced oil recovery (EOR) through low-salinity water flooding (LSWF) or engineered water flooding (EWF) in carbonate reservoirs a subject of intense speculation [1,2]. Carbonate reservoirs are important, as they hold the majority of the proven oil reserves in the world, less than about 30% of which are recoverable [3,4]. This large quantity of unrecoverable oil is attributed to the complex nature of the rock in terms of its heterogeneity, the presence of fractures, and the mixed or oil-wet conditions of the formations [5,6]. The use of nanoparticles (NPs) has been reported to improve oil recovery from carbonates at the tertiary stage [7,8]. This study focuses on investigating the active mechanisms at different temperatures and the effect of temperature on the performance of a hybrid scheme.

### 1.1. Low-Salinity Water Flooding in Carbonate

LSWF has been recently applied to carbonate reservoirs with positive results [6,9]. The process entails modifying the ions of the injection fluid to alter and positively affect the water-oil-rock interactions in the porous media, in order to improve oil recovery via a combination of mechanisms such as changes in wettability, increased pH, multi-ion exchange, etc. [10]. When the ionic strength or the composition of the injection water is tuned, it is known by a specific term, such as low-salinity water (LSW), smart water, smarter water, EW, etc. [11]. The potential benefits of adjusting the composition of injection water in terms of improving oil recovery in an efficient and cost-effective manner have been reported by many researchers [10,11,12,13,14,15,16,17,18,19,20,21,22].

### 1.2. Nanoparticle-Assisted EOR

The world is moving towards the application of nanotechnology to solve numerous problems in different fields. Particles with dimensions ranging between 1 and 100 nm are known as NPs [23]. These are widely accepted as EOR agents due to their unique characteristics, including their small size, which increases the contact angle between the NPs and oil phase and makes the process of oil recovery more effective. Other properties of NPs include low cost, ease of manipulation of the surface to suit porous media conditions, and resistance to harsh conditions in terms of in situ temperature and pressure [24,25,26].

Nanofluids (NFs) can be described as two-phase systems in which a solid phase (NPs) is contained within a liquid phase (dispersant) [27]. NF is prepared by dispersing NPs into a suitable liquid phase at a volume fraction that can form a stable solution [28]. Jaewoo et al. 2019 [29] synthesized Si/SiO_x_ adopting magnesiothermic reduction of silica with different oxygen contents. They concluded that the oxygen content plays a very vital role by affecting electrochemical properties which in turn give better performance.

Ogolo et al. [30] have reported that some common dispersants used for NPs include deionised water, ethanol, mineral oil, and ethylene glycol. Various nanoparticles such as SiO_2_, Fe_2_O_3_, Al_2_O_3_, TiO_2_, and CUO have been applied in an attempt to improve oil recovery over the years [31,32,33]. Since NF consists of two phases, this may pose challenges in terms of stability. The attraction and repulsion forces between NPs play a vital role in the stability of NF. According to the DLVO theory, when the force of attraction exceeds the repulsive forces, sedimentation and agglomeration occur and the particles precipitate, resulting in instability of the fluid. When the force of repulsion exceeds that of the attractive forces, a stable solution is achieved [27].

The stability of NF can be measured by various means, such as sedimentation and centrifugation methods, spectral absorbency analysis, Zeta potential measurements [27] or even visual inspection. A range of factors can affect the stability of NF, such as the preparation method used, the temperature, the salinity of the base fluid, the concentration of the NPs, the size of the NPs, the permeability of the porous media, and the injection rate [8,33,34,35,36,37,38]. The challenge of achieving stability can be addressed by adopting correct preparation procedures and in some cases the use of suitable stabilizers such as polyethylene glycol (PEG), polyvinylpyrrolidone (PVP), and/or adjusting the pH value of the solution [33].

Bayat et al. [37] studied the effects of three different NPs (Al_2_O_3_, TiO_2_, and SiO_2_) on EOR using a limestone sample under different temperature conditions. The transportation of individual NPs was explored, and it was reported that Al_2_O_3_, TiO_2_, and SiO_2_ had adsorption rates of 8.2%, 27.8%, and 43.4%, respectively. The differences in the surface charge were identified as the reason for this adsorption, which translate to a reduction in the contact angle. The NP with the highest tendency to be adsorbed onto the limestone surface gives the strongest effect in terms of reducing the wettability from intermediate-wet to water-wet. These authors concluded that all of the NPs studied had the potential to improve oil recovery via an increase in the number of capillaries, a reduction in the viscosity of the oil, changes in wettability, and a reduction in IFT.

In a similar manner, Moghaddam et al. [39] studied the effects of nine NPs (ZrO_2_, CaCO_3_, CNT type 1, CNT type 2, TiO_2_, SiO_2_, MgO, Al_2_O_3_, and CeO_2_) on oil recovery from carbonate reservoirs by performing different preliminary tests to select the best NPs for spontaneous imbibition (SI), and carrying out core flood experiments. SiO_2_ and CaCO_3_ NPs were selected as the best options. The results obtained for both SI and forced imbibition were related to the alterations in the contact angle. Further recoveries of roughly 8% of the oil initially in core (OIIC) were recorded at a tertiary level of flooding, demonstrating the potential of SiO_2_ NP for oil recovery at the tertiary stage.

Other investigators [8,30,39] have reported an increase in oil recovery beyond that of conventional means via the injection of NF into oil-wet carbonate reservoirs. Most of the NF EOR mechanisms that have been reported involve structural disjoining pressure (SDP) [23,35,40,41], plugging of pore channels [35], viscosifying the injectants and reducing oil viscosity and IFT [42], changes in wettability [31,42,43,44], and preventing the precipitation of asphaltene [26,45]. Young et al. [46] reported the role of anions in the determination of Si/SiO_2_ surfaces water wettability and how the choice of solvents/dispersant can affect the anion rate of reaction.

### 1.3. EW-NF Flooding as a Hybrid EOR

A combination of two or more different methods of EOR is known as hybrid EOR. The aim of a hybrid approach is to recover more oil in an economical way via post-secondary flooding from porous media. Some authors [47,48,49,50,51,52,53] have reported hybrid schemes involving LSW, such as LSW gas flooding, LSW surfactant flooding, LSW polymer flooding, and LSW NF methods. The use of a combination of techniques and the activation of an individual mechanism typically results in a higher oil recovery rate than standalone techniques. Pourafshary and Moradpour [53] reviewed the synergistic effects of different hybrid methods involving LSWF, and concluded that the incremental recovery of up to 30% of the original oil in place can be achieved. Shirazi et al. [33] performed various different experiments and tests to study the synergistic effects of smart water and TiO_2_ NP. They observed positive effects of the hybrid scheme in terms of alterations in wettability and reductions in IFT, which also led to higher oil recovery rates in SI experiments using different EWs and EW-NFs at 60 °C. Hamouda and Abhishek 2019 [54] further reported that hybrid method involving LSW with Silica NP ensure less formation damage in chalk formation.

Pourafshary and Moradpour [55] also carried out an experimental investigation of the performance of hybrid LSW NF on dolomite carbonate rocks. A core flooding experiment showed an increase in oil recovery of 5.4% using LSW NF, due to the activation and initiation of weak mechanisms in the standalone method. From the reviews that have been carried out thus far, it can be concluded that EW-NF flooding is a virgin area of research in the field of EOR that needs to be extensively and carefully explored. Investigations in this area have been scarce, and the few works available have reported promising effects from this hybrid scheme in terms of oil recovery in an economical and environmentally friendly manner.

In the present study, different experiments and tests are performed to investigate the effects of hybrid LSW/SiO_2_ NPs on rock/fluid interactions and oil recovery from carbonates at low and high temperatures. CSW was used as a base fluid for the preparation of EW and EW-NF. Zeta potential measurements were conducted to enable stability analysis and an exploration of the changes at the surface of the rock. A scanning electron microscope (SEM) was used to study both the elemental composition of the rock and that of the NPs used in the study. We performed contact angle measurements, a pH analysis, a study of the rheological behaviour of EW/NF and measurements of IFT, in order to investigate the fluid-fluid and fluid-rock interactions. Finally, a forced imbibition experiment was performed to analyse the impact of temperature on oil recovery via the hybrid method from carbonates.

## 2. Materials and Methods

### 2.1. Materials

#### 2.1.1. Reservoir Rock Sample

**Figure 1:**
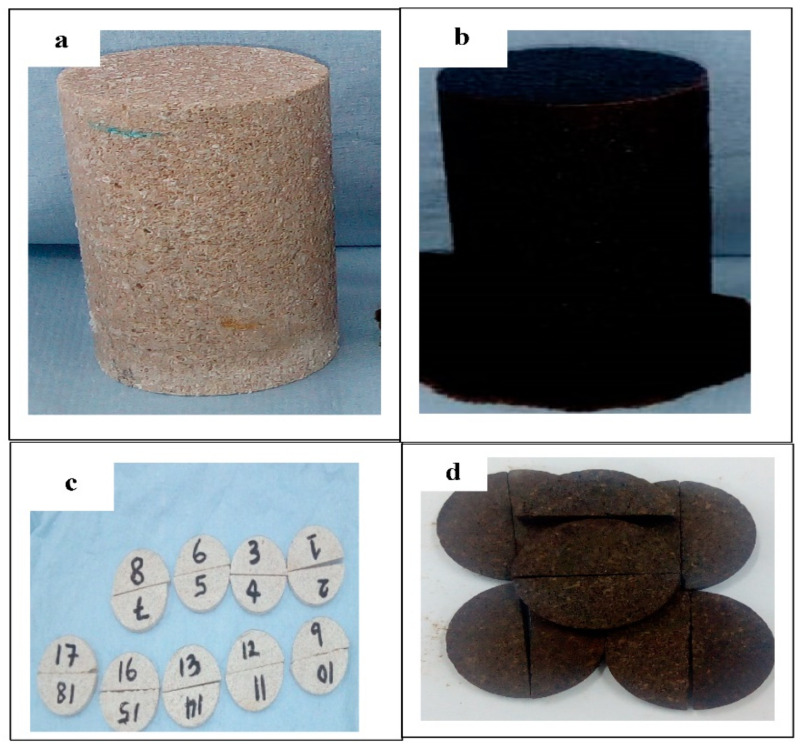
Samples used in the experiments: (**a**) Core plug used in the forced imbibition experiment, before aging in oil; (**b**) core plug used in the forced imbibition experiment, after aging in oil; (**c**) pellets used for contact angle measurements, before aging in oil; (**d**) pellets used for contact angle measurements, after aging in oil.

**Figure 2:**
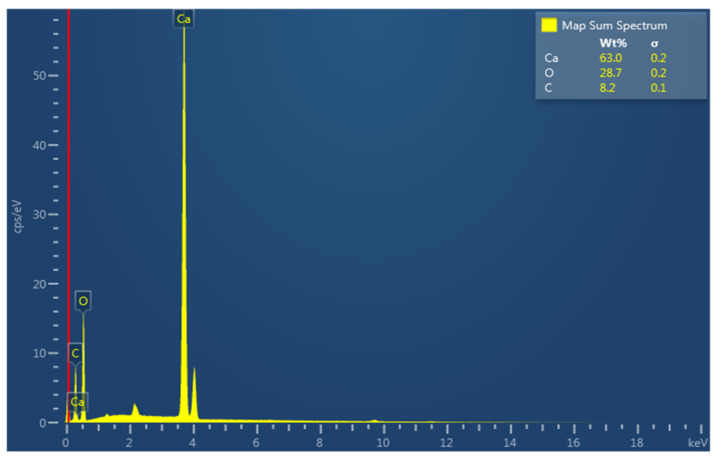
EDS analysis, showing the composition of the rock sample.

**Figure 3:**
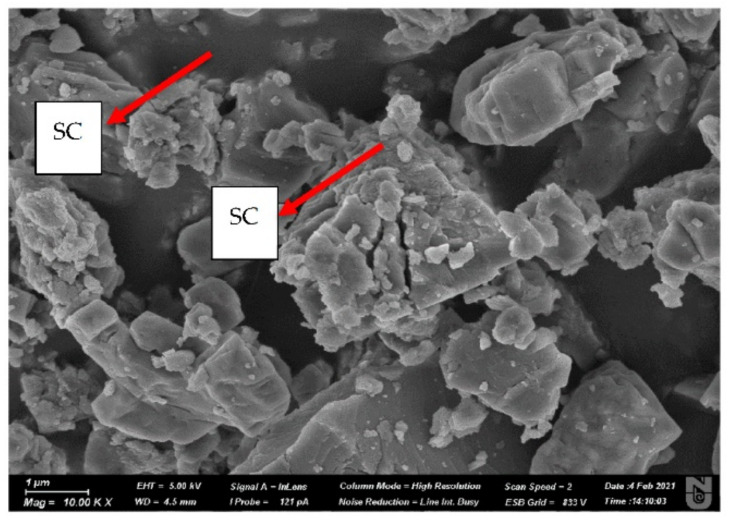
Image of rock sample, taken using a Crossbeam 540 SEM.

Limestone rock samples were used in this experiment. Pellets and a core plug were cut from the rock as shown in Figure 1. An energy dispersive spectroscopy (EDS) analysis of the rock sample is presented in Figure 2, in which the elemental composition of the rock and the type of rock can be seen. Figure 3 shows an image of a rock section obtained using a Crossbeam 540 SEM (Zeiss, Oberkochen, Germany). The high porosity and low permeability of the rock can be observed as a result of the secluded cavity (SC) and a high level of compaction. The properties of the core plug used in the core flood experiment are shown in Table 1.

#### 2.1.2. Crude Oil

**Figure 4:**
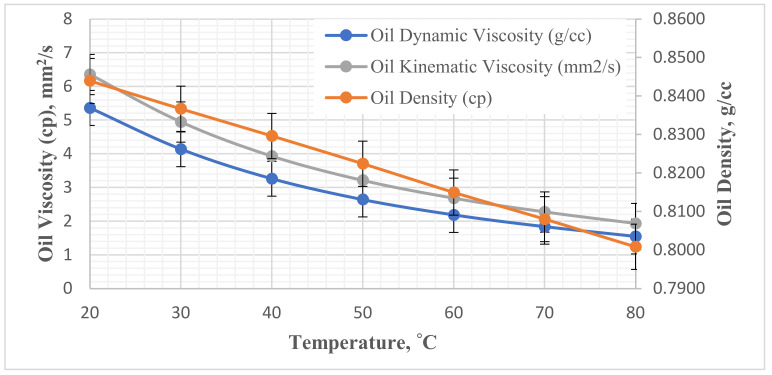
Physical properties of the crude oil sample.

The crude oil sample used in this experiment with 35 American Petroleum Institute (API) gravity was obtained from a Kazakhstani oil field in the Caspian Sea region. The oil was purified via filtration to eliminate impurities such as solid particles and dissolved water and gases. The properties of the oil are shown in Figure 4.

#### 2.1.3. Salts

The various salts used to prepare the brines and the different EWs used for this research were supplied by the Sigma-Aldrich Chemical Company (Missouri, MO, USA). The salts were NaHCO_3_, Na_2_SO_4_, NaCl, KCl, CaCl_2_, and MgCl_2_·6H_2_O.

#### 2.1.4. Brines

The CSW was diluted four times, and is referred to here as 4dsw. The 4dsw was then tuned by adjusting the potential modifying ions, and the monovalent ions were kept constant and unaltered in order to prepare different EW samples to study the effects of the active ions. The details of the design and formulation are shown in Table 2. A four-fold dilution of CSW was chosen as the base fluid based on the results of our previous studies [56]. The salinity of the CSW used in this experiment as an injection/interaction fluid was 13,000 ppm, and formation water (FW) from a field in the Caspian Sea region with a salinity of 183,000 ppm was used as shown in Table 3.

#### 2.1.5. Nanoparticles

**Figure 5:**
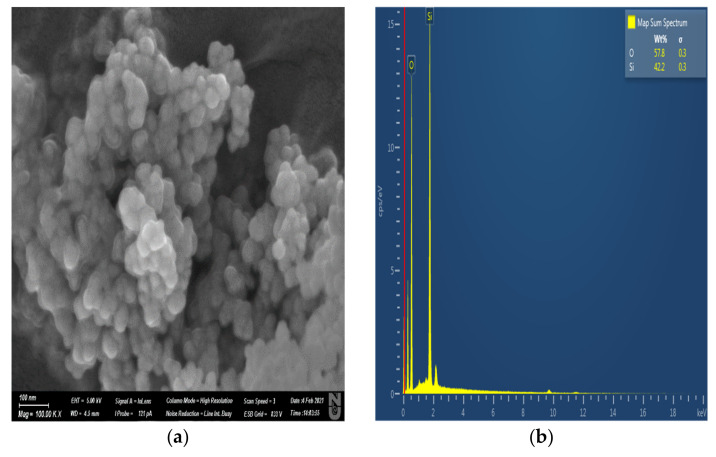
(**a**) Image of SiO_2_ NP taken by SEM; (**b**) elemental composition of SiO_2_ NP.

Silicon IV oxide NPs with a size of less than 50 nm and a purity of 99.5% were used in this research. An image of a particle taken using a Crossbeam 540 SEM is shown in Figure 5. The NPs were supplied by SkySpring Nanomaterials (Houston, TX, USA). 

### 2.2. Experimental Procedure

#### 2.2.1. Measurement of the Properties of Brine

In order to appropriately characterise the EW formulations, an Anton Paar SVM 3001 viscometer (Anton Paar, Austria) was used to measure the viscosity and density at different temperatures. It was very important to determine the parameters accurately at this stage, since the density of the EW is required for the correct determination of the contact angle and IFT. All the experimental measurements were within the range of acceptable experimental errors.

#### 2.2.2. Pellet Preparation for Contact Angle Measurement

The prepared pellets were dried in an oven for 48 h, and then saturated for 2 days at a pressure of 1000 PSI. The pellets were aged in crude oil in an oven at a temperature of 120 °C for 8 weeks to attain an initial oil-wet state.

#### 2.2.3. Preparation of the Nanofluids

A range of different EW samples were used as the base fluid. Weighted SiO_2_ particles were dispersed in the brine and stirred for 30 min using a magnetic stirrer at 650 rpm, after which the solution was homogenised using a 4.3-inch thin-film transistor (TFT) homogeniser sonicator for 45 min at a 70% power rating with a frequency of 20–25 Hz. Different concentrations of nanoparticles (0.05, 0.1, 0.2, 0.3, 0.4, and 0.5 wt%) were prepared.

#### 2.2.4. Measurement of the Zeta Potential

A Malvern Zetasizer Nano ZSP (Malvern, United Kingdom) was employed to measure the zeta potential of each sample. Based on the values recorded, the stability of each fluid was established by comparing it with a standard stability indicator (Table 4). The zeta potential measurements showed that all of the EW NFs could be considered stable. It is well-known that EW NFs with twice and four times SO_4_^2−^ ions are very effective hybrid fluids in terms of changing the surface charge and improving the detachment of oil from the rock surface.

#### 2.2.5. Contact Angle Measurements

A DataPhysics Optical Contact Angle System OCA 25 (DataPhysics, Filderstadt, Germany) was used to measure the contact angle for the pellets before aging in oil, using the captive bubble method [57]. The pellets were found to be completely water-wet with an average value of 0.5°, and were then soaked in oil and aged in an oven for 8 weeks at 120 °C. The contact angles were measured after aging to verify the oil-wet condition of the samples. Three different measurements of the contact angle were made from both sides of each pellet, and the average values were reported. All measurements were made both at a reduced temperature and an elevated temperature of 80 °C.

#### 2.2.6. IFT Measurements

EW samples with a range of salinities from 3250–4500 ppm, formation brine with a salinity of 183,000 ppm, and EW-NF solutions containing 0.1 wt% NP were prepared for the IFT measurements. An OCA 25 EC video-based optical instrument DataPhysics, Filderstadt, Germany) was used to evaluate the IFT for the oil and prepared fluids using the pendant-drop method. Measurements were taken both at room temperature and under high-temperature conditions.

#### 2.2.7. The pH Measurement

A pH strip was used to measure the pH of each EW and EW-NF, before and after the treatment of the pellets.

#### 2.2.8. Rheology Measurement

The rheology of EW-NFs was measured under different shear rates at a reduced temperature of 25 °C and an elevated temperature of 80 °C, using an MCR 302 modular compact rheometer (Anton Paar, Austria).

#### 2.2.9. Energy Dispersive Spectroscopy Analysis

A Crossbeam 540 SEM was used to capture images of the pellets soaked in EW and then later soaked in EW-NF. The aim of this was to investigate the degree of attachment of SiO_2_ NP and polar oil components to the rock surface, and to analyse the possible ion exchange.

#### 2.2.10. Core Flooding Experiment

**Figure 6:**
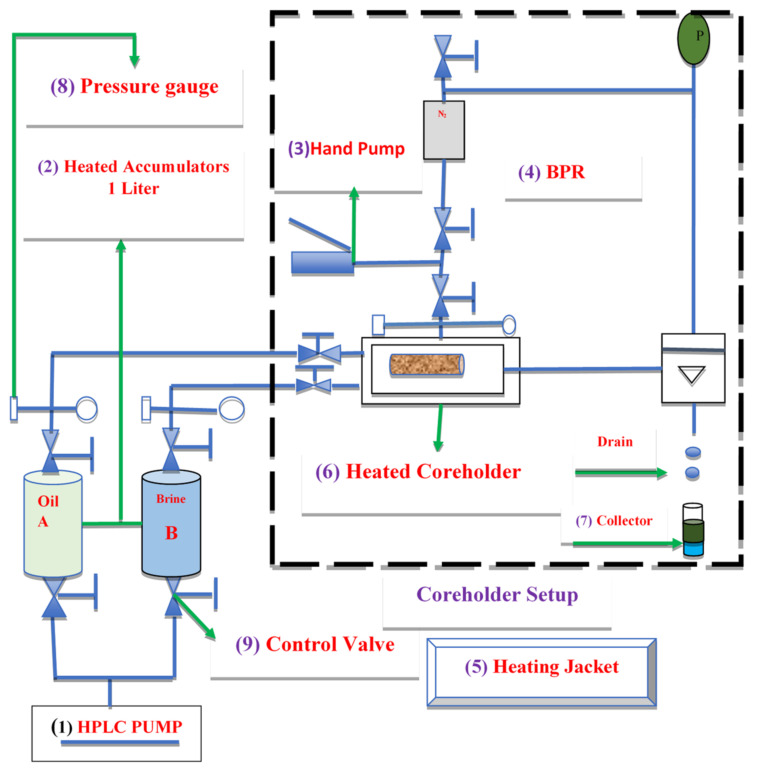
Schematic diagram of the core flood setup.

The components of the core flood apparatus setup were as follows: (1) An injection pump; (2) two sets of accumulators; (3) a pump for applying confining pressure; (4) a back pressure regulator; (5) heating jackets to keep the fluid in the accumulator and the sample in the core holder at the desired temperature; (6) a core holder; (7) a collector to gather and measure the effluent extracted from the core; (8) a pressure gauge; and (9) control valves, as shown in Figure 6.

The core plug was dried in an oven for 24 h at a temperature of 80 °C. It was then saturated with a saturator for another 24 h at a pressure of 1100 Psia. The core plug was then flooded with brine to ensure it was completely water-wet. Oil was injected until no more water was produced from the core, in order to establish irreducible water saturation. The core was then aged in an oven for 1 month at a temperature of 120 °C to achieve a fully oil-wet state. It was loaded into the core holder at a confining pressure of 1050 Psia and a back pressure of 500 Psia. EW was first used for flooding, and this was followed by EW-NF, until no more oil was recovered. The injection rates were 0.5, 2, 5, and 7 cc/min. This process was designed to mimic the field injection rate and to avoid any capillary end effects. The amount of oil recovered at each injection stage was measured and reported. 

## 3. Results

This section may be divided by subheadings. It should provide a concise and precise description of the experimental results, their interpretation, as well as the experimental conclusions that can be drawn.

### 3.1. Density and Viscosity Measurements of Brines and EW-NF

**Figure 7:**
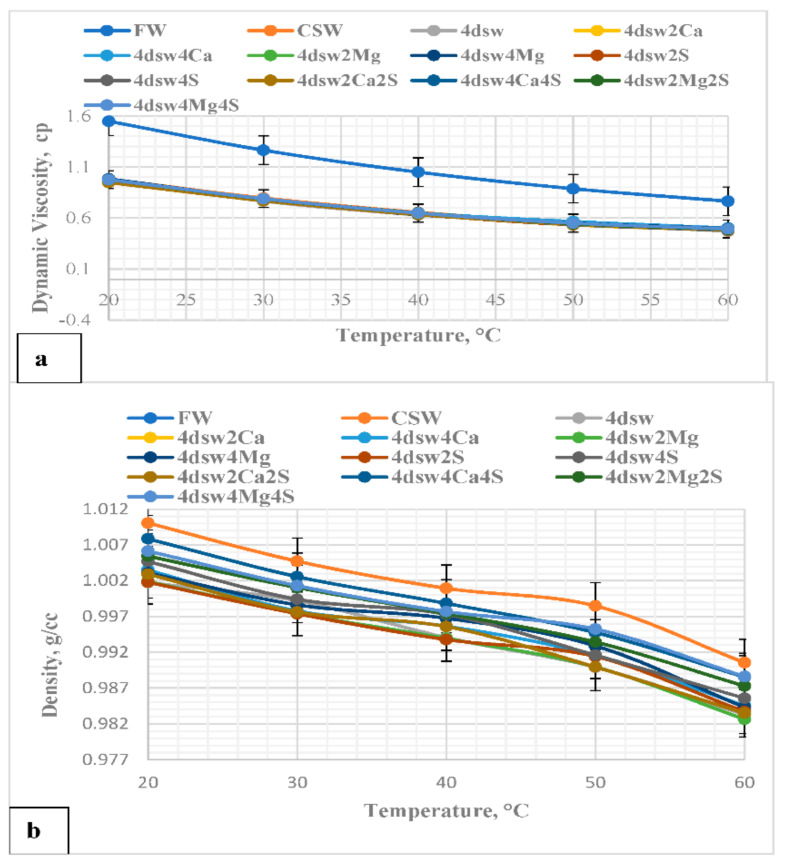
(**a**) Viscosity-temperature relationships for the FW and EW samples; (**b**) density-temperature relationships for the FW and EW samples.

**Figure 8:**
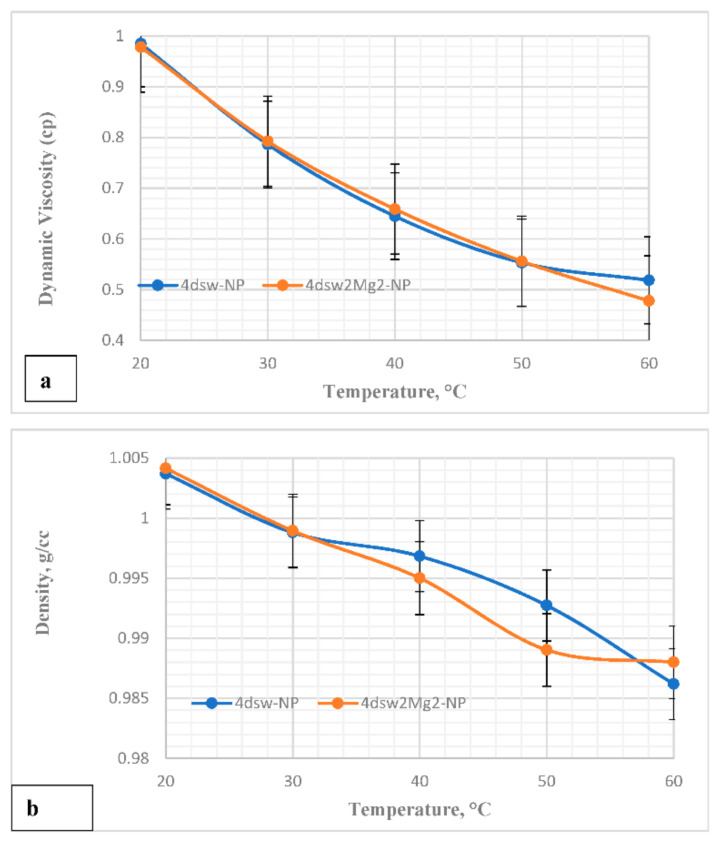
(**a**) Viscosity-temperature relationships for EW-NF; (**b**) density-temperature relationships for EW-NF.

Figure 7 shows a plot of the density and viscosity of the EWs at different temperatures. It can be seen from Figure 7a that the viscosity of the FW was slightly greater than that of the EWs, and this was due to the higher total proportion of dissolved solids in the FW. The viscosities of all the EWs were similar, since they had comparable ionic strength and total proportions of dissolved solids. It can be clearly observed that the density and viscosity of each EW was reduced as the temperature increased. The relationships between brine density, viscosity, and temperature were nonlinear [58]. Figure 8 shows a similar trend for the EW-NF fluids.

### 3.2. Stability of the Nanofluids

One of the major factors that affect the stability of NFs is the preparation time, and in order to mitigate its effect, a homogenisation time of 45 min was adopted based on the results of our previous study [56]. Zeta potential measurements and visual inspection were used to study the effect of salinity on the stability of NFs. An increase in salinity reduces the stability of NF, as the ions present in the saline medium neutralise the surface charge of the NPs [33]. An unstable fluid should not be injected into the porous media, in order to avoid problems related to permeability [8,33,36]. A salinity range of up to 5000 ppm is reported to be suitable as a base fluid for the preparation of SiO_2_ NF [56]. A concentration of 0.1 wt% NP was selected as the optimum concentration in this experiment [31,56].

### 3.3. Rheology of the Nanofluid at Different Temperatures

**Figure 9:**
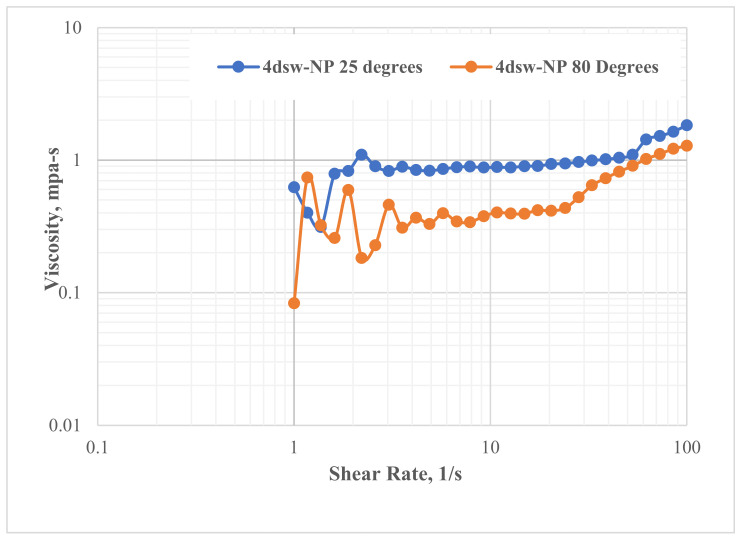
Rheological behaviour of EW-NF.

The rheological behaviour of different EW-NFs was measured at both the ambient temperature and an elevated temperature of 80 °C, using a rheometer. Figure 9 shows an example of the effect of temperature on the viscosity of LSW NF sample. A slight decrease in the viscosity of the fluid with increasing shear rate can be observed at all temperatures. This is an indication of a shear thinning behaviour that follows a power law model. As the shear rate increases, the trend changes to shear thickening behaviour, irrespective of the temperature; this is an indication of a good mobility parameter that ensures favourable oil displacement from the porous media. Further details can be found in our recent paper [56].

### 3.4. Contact Angle Measurements

The interactions between the hybrid fluid, oil, FW, and rock were studied via contact angle measurements. The fluid-fluid interaction was also investigated through interfacial tension measurements. Both types of measurements were taken at a reduced temperature and an elevated temperature of 80 °C, to allow us to analyse the effect of temperature on the rock/fluid interactions. The impacts of both the EW and NF samples were analysed in order to select the best fluid for further tests.

### 3.5. Effects of Hybrid Fluids on Wettability

Pellets were strongly oil-wet at the end of the aging process, due to the high level of acidity of the oil. The treatment of the oil-wet pellets was carried out under two different conditions: Room temperature and an elevated temperature of 80 °C. The experiments under the first condition were carried out in three stages. In the first stage, the pellets were soaked in different EW solutions for 72 h to study the effect of the standalone EW interaction on changes in wettability. In the second stage, the pellets were soaked in EW-NF for 48 h, and in the final stage, the pellets were re-soaked in EW-NF for one additional week. In the experiments under the second condition of elevated temperature, the treatment of the pellet was carried out in four stages, as summarised below:

1. The pellets were soaked in different EW solutions for 24 h in an oven, in order to study the effect of a standalone EW interaction on changes in wettability at a reservoir temperature of 80 °C.
2. The pellets were soaked in a range of different EW-NFs for an additional 24 h to study the effects of hybrid fluids on CRB interactions.
3. The pellets were re-soaked in the same EW-NF for another 24 h.
4. Step 3 was repeated for another 48 h.

The main aims were to study the effects of temperature on changes in wettability due to the application of the hybrid approach, and to compare the results with those obtained at the ambient temperature.

#### 3.5.1. Effect of Dilution

**Figure 10:**
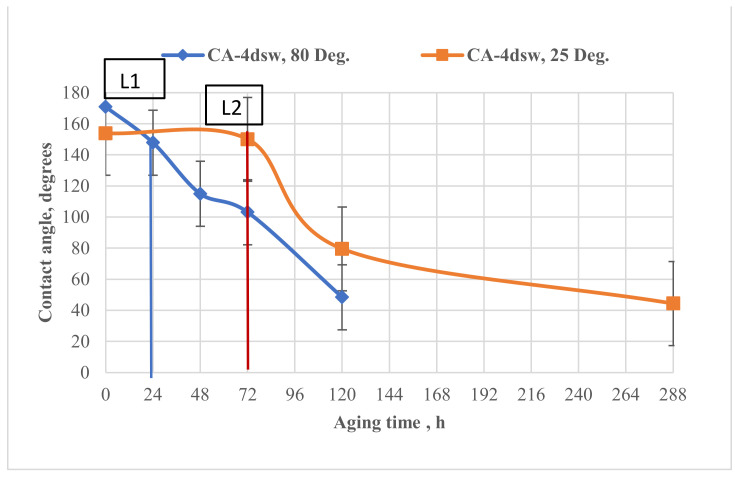
Effect of dilution on the contact angle, at ambient temperature and an elevated temperature of 80 °C.

**Figure 11:**
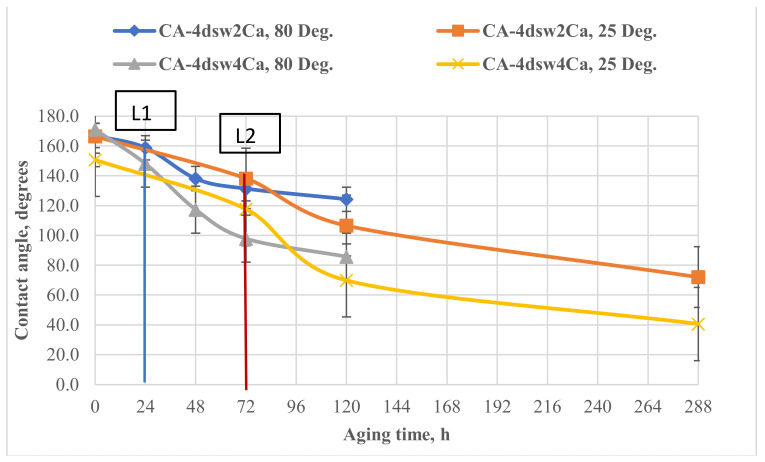
Effect of active ions (Ca^2+^) on the performance of EW and EW-NF, at room temperature and an elevated temperature of 80 °C.

**Figure 12:**
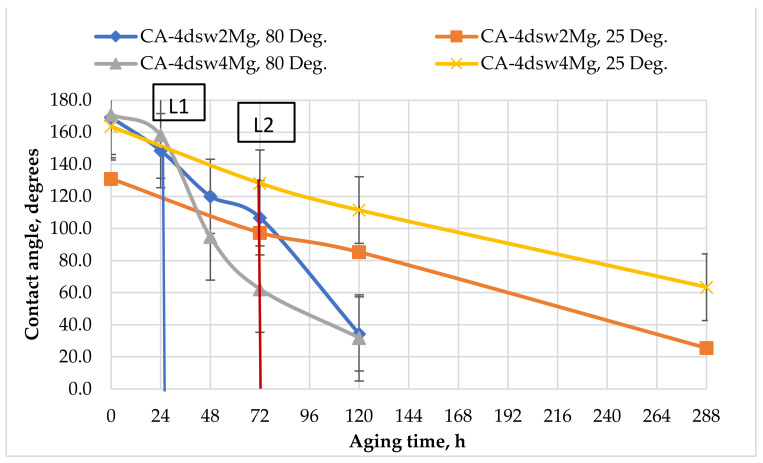
Effect of active ions (Mg^2+^) on the performance of EW and EW-NF, at room temperature and an elevated temperature of 80 °C.

**Figure 13:**
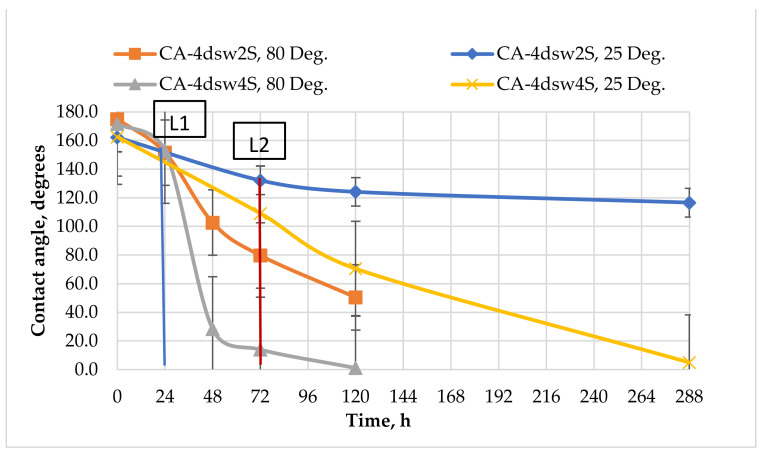
Effect of active ions (SO_4_^2−^) on the performance of EW and EW-NF, at room temperature and an elevated temperature of 80 °C.

**Figure 14:**
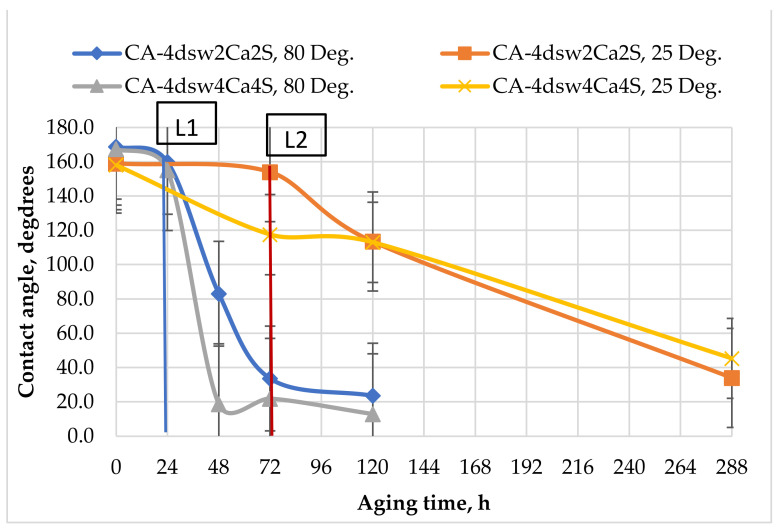
Effect of active ions (Ca^2+^ and SO_4_^2−^) on the performance of EW and EW-NF, at room temperature and an elevated temperature of 80 °C.

**Figure 15:**
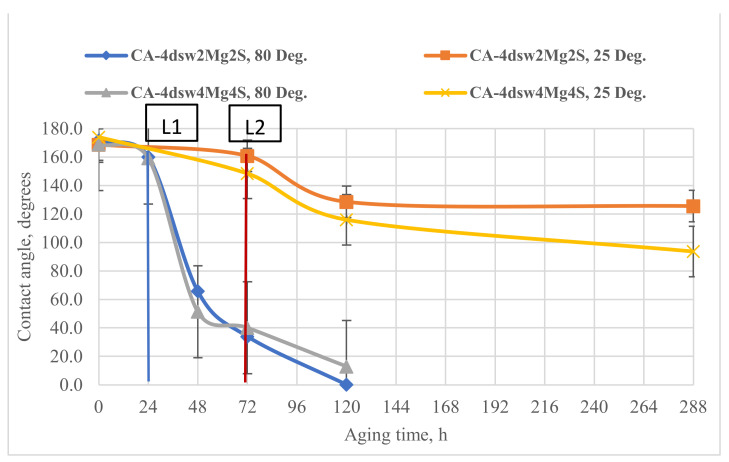
Effect of active ions (Mg^2+^ and SO_4_^2−^) on the performance of EW and EW-NF, at room temperature and an elevated in situ temperature of 80 °C.

Figure 10 shows the effect of dilution of the CSW and EW-NF on the oil-wet pellets, at both room and elevated temperatures. The labels L1 and L2 used in Figure 10, Figure 11, Figure 12, Figure 13, Figure 14, Figure 15 represent the end of EW treatment and the beginning of the EW-NF treatment of a pellet at elevated in situ and reduced ambient temperatures, respectively. The main aim of this process was to compare the rate of change of wettability due to EW and EW/NF at low and high temperatures.

Figure 10 shows that soaking the oil wet pellet in 4dsw at low temperature caused a change in the contact angle of only 4° after 72 h. Applying the hybrid method accelerated the process and made it more effective by altering the wettability from a strong oil-wet state to an intermediate wetting condition, and finally to a strong water-wet state after 288 h of soaking. 

When the pellets were soaked in EW for 24 h at high temperature, the change in the wettability was faster and its magnitude was a factor of 23 as compared with a factor of four under ambient temperature conditions. Further treatment with LSW NF resulted in a change in the wettability towards an intermediate wet and finally a strong water-wet state. Under ambient temperature conditions, longer contact and a longer treatment time were required to reduce the contact angle by a reasonable factor, while at an elevated temperature, the treatment time was shorter. Therefore, temperature plays a vital role in accelerating the wettability alteration process in carbonates, for both the EW and EW-NF solutions.

#### 3.5.2. Catalytic Effects of Temperature and Active Ions on the Performance of Hybrid Fluids

Figure 11, Figure 12, Figure 13, Figure 14 and Figure 15 show the effects of hybrid EW-NF on the rate of change of wettability. The EW solutions contained different combinations of active ions.

A suitable adjustment and modification of the concentrations of active ions in the EW positively impacts the interaction between the EW and fluid/rock in the porous media. The change in wettability from the EW-NF arises from the ion exchange between the carboxylic group in the oil, divalent cations, and the sulphate ion, coupled with the presence of NPs that sit on the surface of the rock, and then detach and peel off the oil from the surface.

When the pellet was treated with EW at a reduced temperature by soaking in diluted sea water containing twice and four times as many calcium ions at the ambient temperature for 72 h (Figure 11), the reduction in the contact angle was insignificant, thus confirming the inactivity of the active ions at a lower temperature. Further treatment with EW-NF for 216 h eventually shifted the wettability towards water-wet due to the efficiency of the change in wettability arising from the SiO_2_ NPs. The effect of temperature on the calcium ions was also demonstrated, as it was found that the treatment time needed to achieve this change in wettability was shorter at a high temperature, despite the fact that the number of active calcium ion sites on the carbonate surface is reduced at higher in situ temperature. It can be concluded that an elevated in situ temperature significantly affects the treatment time.

Raising the concentration of Mg^2+^ in diluted sea water by twice and four times reduced the contact angle at almost the same rate at ambient temperature, as shown in Figure 11. The presence of the NF did not affect the rate of change of wettability, as Mg^2+^ ions are generally not very effective at low temperatures [59]. Under high-temperature conditions, increasing the concentration of Mg^2+^ in LSW by twice and four times reduced the wettability by 23 and 13°, respectively, within 24 h of soaking. Further treatment with EW-NP reduced the CA to around 32°, giving a very strong water-wet condition. This observation confirms the catalytic effect of temperature on the magnesium ion in the presence of NPs, in terms of accelerating the wettability alteration process in a carbonate reservoir.

Figure 13 illustrates the ability of the divalent anion (sulphate) to reduce wettability. Increasing the concentration of the sulphate ion in 4dsw by four times, both in EW and EW-NF, gave a substantial reduction in contact angle compared with twice sulphate ion spiked in 4dsw as EW and EW-NF, in which the wettability was not altered to even intermediate-wet even after 288 h of soaking and treatment. However, the catalytic effect of temperature could be observed at both twice and four times the concentration of the sulphate ions in the presence of NPs, and the wettability was shifted towards a strong water-wet condition within only 24 h of treatment for a four-fold increase in concentration. The rate of change in the wettability of EW/EW-NF with four times the concentration of sulphate ions was higher than for twice the concentration, at both the ambient and elevated in situ temperatures. Hence, sulphate ions in the presence of NPs at an elevated temperature show a stronger catalytic effect in terms of altering and shifting the wettability to a more favourable state in carbonates.

The effects of combining active cations with sulphate ions in the presence and absence of NP at ambient temperature and a high temperature of 80 ℃ are illustrated in Figure 14 and Figure 15. The same effect was observed, in that the use of the hybrid method at a higher temperature was very effective in accelerating the wettability alteration process. At a lower temperature, we recommend using a combination of Ca^2+^ and SO_4_^2−^ ions in the hybrid method, due to the stronger activity of calcium cations compared to magnesium. The combined effects of the high temperature and the presence of the cations and SO_4_^2−^ active ions in the hybrid fluid means that the wettability alteration process is much faster at 80 °C, and the contact angle is changed to a very low value within only 24 h of treatment.

**Figure 16:**
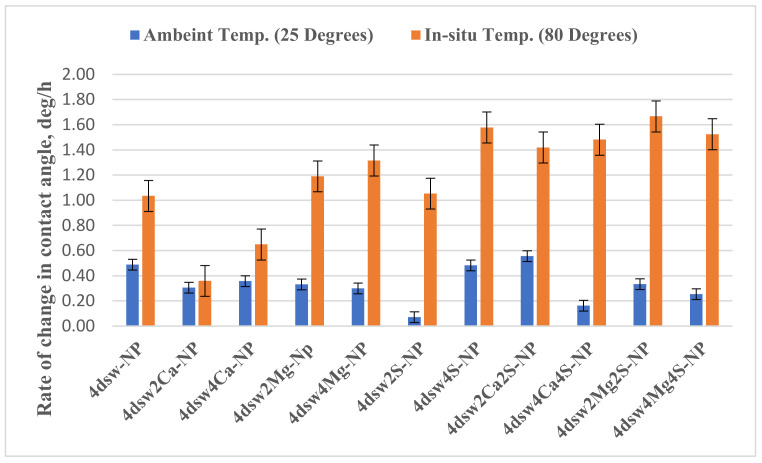
Effect of temperature on rate of change of wettability by EW-NF.

Table 5 and Figure 16 show the effects of temperature on the rate of change of wettability in carbonate rock, for both the standalone EW and the hybrid EW-NF methods. It can be seen from Figure 16 that the performance of all of the active ions is higher at the high temperature. It can be concluded that temperature does not change the effectiveness of the calcium ions but is critical for the effectiveness of magnesium. Hence, at higher temperatures, the use of the Mg^2+^ active ion is recommended. The presence of sulphate is very important at high temperature. Therefore, we recommend using SO_4_^2−^/Mg^2+^ in the hybrid method at high temperatures, and SO_4_^2−^/Ca^2+^ at low temperatures.

**Figure 17:**
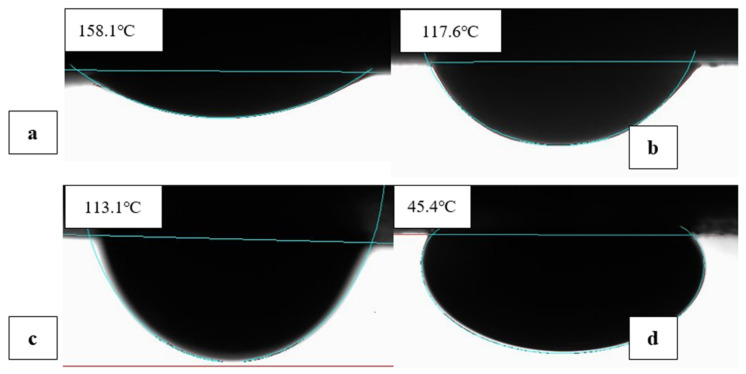
Effect of EW-NF on pellet treatment at reduced temperature: (**a**) The initial oil-wet state; (**b**) after soaking in 4dsw2Mg2S for 72 h; (**c**) after soaking in 4dsw2Mg2S-NP for 48 h; and (**d**) after soaking in 4dsw2Mg2S-NP for an additional week.

**Figure 18:**
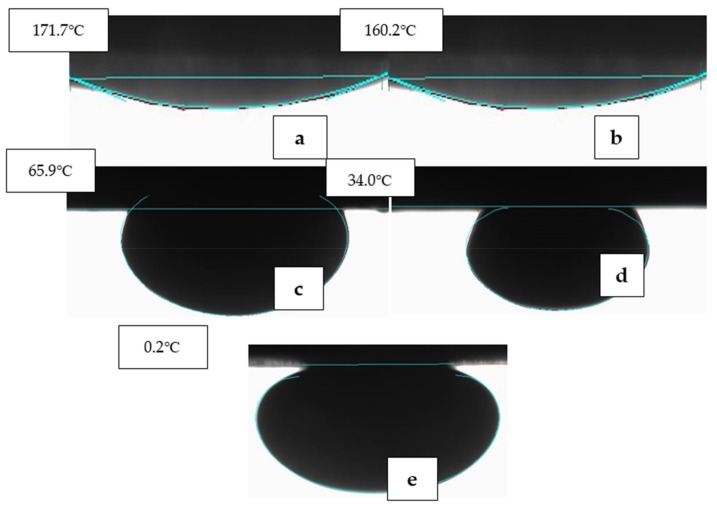
Effect of EW-NF on pellet treatment at elevated temperature: (**a**) The initial oil-wet state; (**b**) after soaking in 4dsw2Mg2S for 24 h; (**c**) after soaking in 4dsw2Mg2S-NP for 24 h; (**d**) after soaking in 4dsw2Mg2S-NP for a further 24 h; (**e**) after soaking in 4dsw2Mg2S-NP for a further 48 h.

Figure 17 and Figure 18 show the stages of wettability reduction by one of the optimised EW-NFs at ambient and elevated temperature, respectively. Both the individual and combined active ions that were ineffective at a low temperature, despite a long soaking period (Figure 17), were activated at high temperature, and the contact angle was significantly reduced within shorter soaking and treatment times (Figure 18). Therefore, the temprature plays a vital role in the effectiveness of the hybrid method and accelerates the production from carbonate rocks.

**Figure 19:**
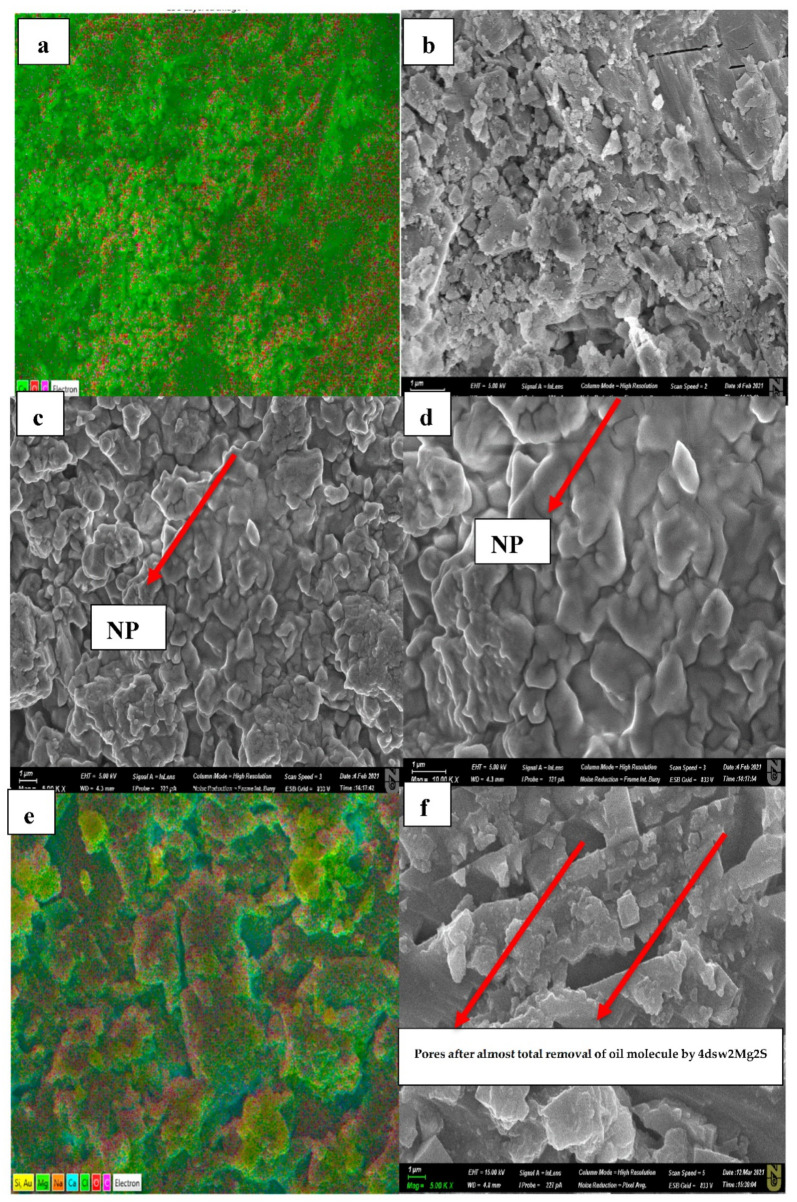
(**a**,**b**) SEM images of newly cut limestone; (**c**,**d**) deposition of SiO_2_ on the oil-wet rock surface after soaking in hybrid LSW/NF; (**e**,**f**) deposition of different ions and NPs after total removal of oil from the rock surfaces by EW-NF.

Figure 19 shows SEM images of the aged rock samples used in the experiment. It can be seen that different ions and nanoparticles were deposited on the surface during each aging process. These ions and particles were further analysed to gain a better understanding of the key mechanisms underlying the effective and catalytic oil recovery via the hybrid method.

Figure 19a,b shows the carbonate rock before aging, which contains only the elements Ca, C, and O, as per the chemical formula for limestone (CaCO_3_). Figure 18c,d shows aged pellet after soaking in EW-NF solution. It can be seen that NFs cover both the salt crystals and the oil components deposited on the rock surface during soaking in EW and subsequent aging in oil at an elevated temperature in an oven. Figure 18e,f shows that the oil completely detaches from the surface of the rock, and different ions (Mg^2+^ shown in green, Ca^2+^ in blue) and NPs (shown in yellow) are deposited on the rock. The active mechanism here can be described as an alteration in wettability via multi-ion exchange between the rocks, and both EW and NPs are involved in this interaction process. SDP is also generated, which weakens the adhesion forces of the oil droplets and finally peels off the oil from the rock surface. Virtually all the EW-NFs showed a similar trend to that in Figure 18f. In order to investigate the effects of temperature on the performance of the Mg^2+^ ion on oil recovery in carbonates, LSW NF with twice the concentrations of Mg^2+^ and SO_4_^2−^ ions was used for the core flood test.

### 3.6. Interfacial Tension

Measurements of crude oil EW and crude oil EW-NF at room temperature did not show a significant reduction in IFT, meaning that the effect of EW and NF on IFT was negligible. However, at 80 °C, a significant reduction in IFT between oil and EW was observed. The IFT of the LSW NP solution was also reduced further by 2 mN/m. This reduction is not significant enough to give a reasonable recovery rate using a standalone EOR technique but can make a meaningful impact in a hybrid EOR such as the EW-NF method when applied to carbonate. Increasing the temperature can therefore activate a reduction in IFT as a weak mechanism of enhancing oil recovery. This mechanism becomes much weaker at low temperatures.

### 3.7. Core Flood Experiment

**Figure 20:**
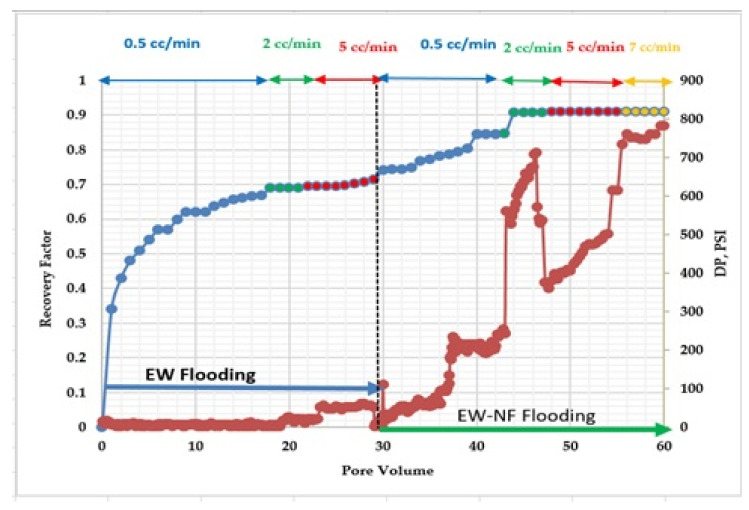
Results for oil recovery from EW and EW/NF hybrid flooding.

The main aim of this test was to investigate the effects of the optimised LSW NF on oil recovery at an elevated temperature. A core flood was conducted by firstly injecting EW until no further recovery was recorded, followed by the EW-NF injection until no more oil could be produced and recovered from the core. Figure 20 shows the results for the recovery from the core flood test versus the pore volume injected, together with the pressure profile for the injection process. EW was found to be very effective, recovering up to 71% IOIC, while EW-NF recovered an additional 20% of the OIIC, an indication of the positive effects of the hybrid approach compared to the standalone techniques of EW injection or NF. A further analysis of the oil saturation profile showed that the residual oil was reduced to the minimum possible level. A mobility estimation indicated a very favourable displacement and delay in water breakthrough. The analysis of the coreflood experiment indicated that the higher rate of wettability alteration and improved oil recovery are achievable through the adjustment of PDIs of the injection brine. Furthermore, the temperature was discovered to speed up the rate of wettability change from oil wet to mix/water wet condition that improves recovery in carbonate formations.

**Figure 21:**
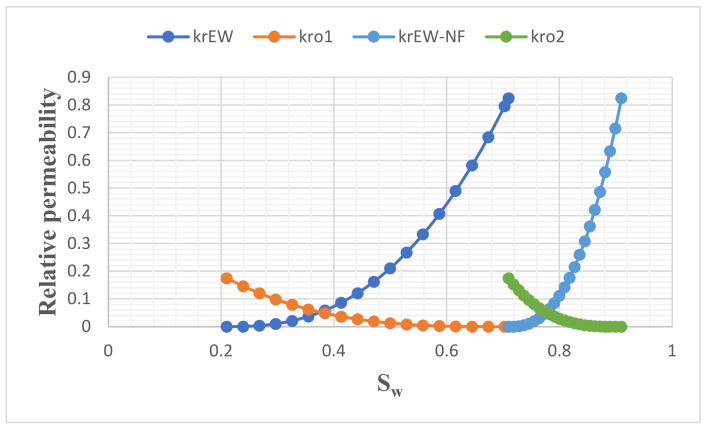
Relative permeability curves for EW and EW-NF flooding.

**Figure 22:**
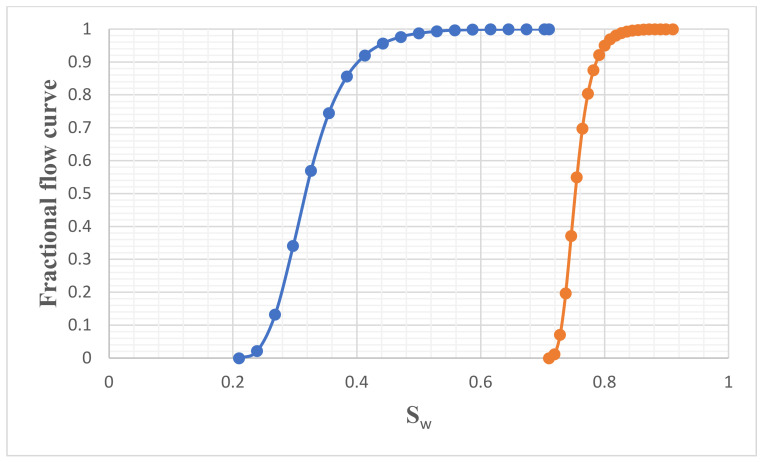
Fractional flow curves for EW and EW-NF.

Despite the low permeability of the core plug, great success was achieved with this hybrid approach. The relative permeability and fractional flow datasets were generated with the Corey model [60], and graphs of these results are shown in Figure 21 and Figure 22, respectively. It can be seen from Figure 21 that the highest relative permeability to oil was 0.175, and the corresponding value for water was 0.825. The relative permeability curve was shifted to the right, which indicates a change in wettability towards water-wet and translates to a high rate of recovery by the EW-NF by reducing the residual oil saturation to the minimum allowed by the capillary forces. The fractional flow curve also indicated a favourable displacement and delay in water breakthrough (Figure 22).

### 3.8. Active Mechanisms of EW/EW-NF Flooding at Different Temperatures

**Figure 23:**
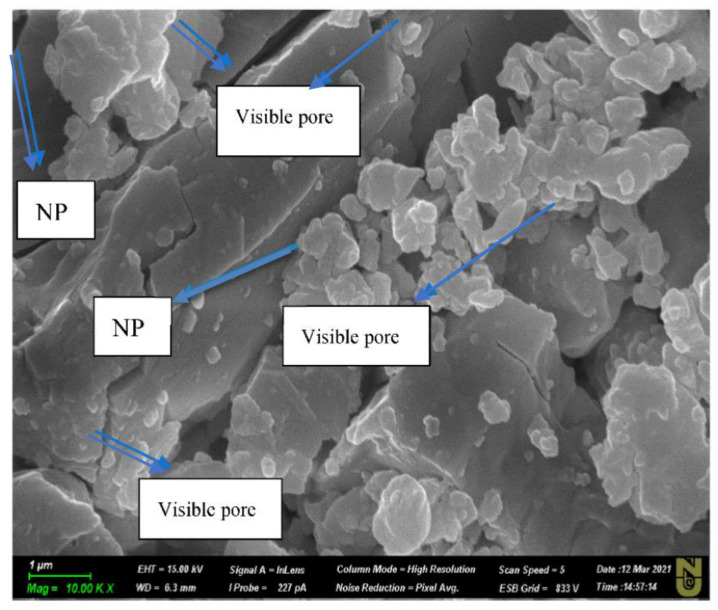
Image of a core sample after the flood test.

The active mechanisms underlying the EOR hybrid method were studied via a range of different experiments. Figure 19d shows how silicon oxide NPs and active ions from the EW-NF solution filled the sites on the soaked pellets. The excellent performance of the hybrid approach can be observed in Figure 19f, in which the pore and crystal of the rock can be clearly seen after aging in the EW-NF solution. Figure 23 shows an SEM image of rock cut from the center of the core plug after completion of flooding. It can be seen that NPs sit on the rock surface and completely remove the oil from the pore spaces. This effect explains the recovery of an additional 20% OIIC from the core flood test. It can be observed from Figure 11, Figure 12, Figure 13, Figure 14 and Figure 15 that the combined effects of the hybrid scheme in terms of reducing wettability are much stronger at the higher temperature than at the lower temperature, and give much faster results. Multi-ion exchange between the EW and the rock surface, adsorption of NP onto the rock surface, and the SDP are therefore catalytically initiated and more strongly activated at a higher temperature than a lower one, which gives a very slow process.

**Figure 24:**
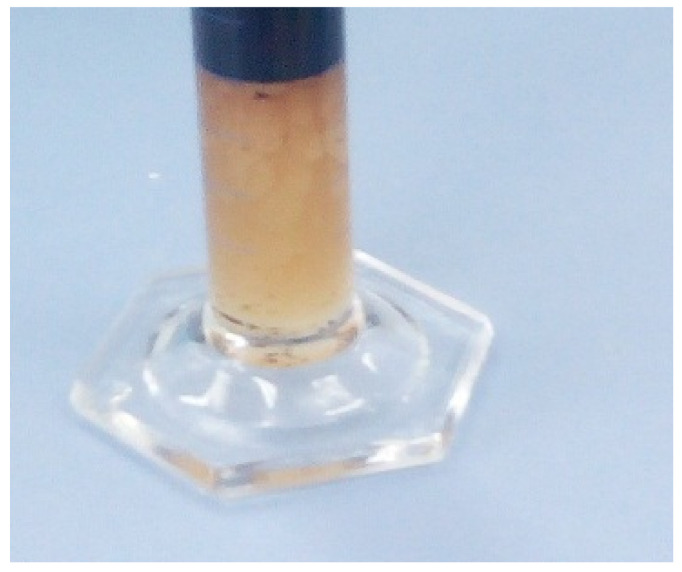
Microemulsion formation.

Furthermore, the pressure profile was stable during the injection of EW, and an increase was observed when EW-NF was used as the injection fluid. The pressure drops increase as a result of the activation of another mechanism called microemulsion formation (Figure 24), which arises due to the presence of NPs. The development of microemulsions leads to an increase in the viscosity of the injectant and ensures favourable mobility. A catalytic effect of temperature is also observed due to the reduction in IFT at a higher temperature. Weak mechanisms in standalone EOR are therefore activated when the hybrid method is applied at a high temperature.

## 4. Conclusions

Several studies have been conducted on hybrid methods comprising LSWF/EWF and other EOR methods, which in most cases have been beneficial in terms of EOR. However, the effects of temperature on a hybrid EW-NF scheme applied to carbonates have not been extensively explored. In this research work, a range of different EWs were designed to investigate the effects of dilution and the presence of PDIs on the stability of SiO_2_ NFs and the effective rock/fluid interaction parameters such as the wettability, IFT, and rheology, at both the ambient temperature and an elevated temperature of 80 °C. The following conclusions can be drawn based on our findings.

All of the hybrid fluids used in the tests were stable and were therefore suitable for EOR processes. No noticeable change in the IFT between oil and the different EWs or EW-NF was observed at room temperature. However, a significant reduction in IFT was observed at the higher temperature. This confirms that SiO_2_ NPs play an active role in reducing IFT in the hybrid scheme at high temperature.

A suitable modification of the cation concentration in the EW-NF positively affected the wettability. This can be attributed to the interaction of cations with the adsorbed carboxylic groups, which caused desorption and peeled off the acidic group from the surface of the carbonate. Sulphate ions showed great potential in altering the wettability at the surface of carbonate rock, and a stronger effect was observed in the presence of NPs at the higher temperature. Ca^2+^ and Mg^2+^ ions had less effect than sulphate ions in terms of changing the wettability at the lower temperature. However, the Mg^2+^ ions were activated at high temperature, and were found to be very effective in terms of reducing the wettability of carbonate rocks via a multi-ion exchange mechanism and multiple other mechanisms that acted to speed up the wettability process. A combination of divalent active ions and divalent anions was very effective in changing the wettability, which in turn led to a high oil recovery rate at both the secondary and tertiary stages. Virtually all EWs with a combination of ions reduced the wettability of the carbonate rock samples from strong oil-wet to strong water-wet, within a shorter period than at the ambient temperature. It can therefore be concluded that temperature played a catalytic role in changing the carbonate rock surface from a hydrophobic state to the desired hydrophilic one, and an acceleration in the production from carbonate was therefore observed during the flooding process. Finally, in order to achieve the best results from the hybrid scheme, it should be noted that SO_4_^2−^/Mg^2+^ is recommended for the design of EW-NFs at high temperatures, while at a lower temperature, SO_4_^2−^/Ca^2+^ yields excellent performance and improves the recovery rate from the carbonate reservoir.

## Figures and Tables

**Table 1:** Dimensions and physical properties of the core plug.

Physical Properties	Weight (g)	Length (cm)	Diameter (cm)	Porosity (%)	Absolute Permeability (mD)	Effective Permeability to Oil (mD)	S_wi_ (%)	PV	OIIC (cm^3^)
-	205.68	7.96	3.81	14	30	5.3	21	12.54	10.04

**Table 2:** Notation used for EW samples.

ID	Empirical Formulation	Molecular Formulation
1	4dsw	4 × diluted sea water
2	4dsw2Ca	4dsw + 2Ca^2+^
3	4dsw4Ca	4dsw +4Ca^2+^
4	4dsw2Mg	4dsw + 2Mg^2+^
5	4dsw4Mg	4dsw + 4Mg^2+^
6	4dsw2S	4dsw + 2SO_4_^2−^
7	4dsw4S	4dsw + 4SO_4_^2−^
8	4dsw2Ca2S	4dsw +2[Ca^2+^ + SO_4_^2−^]
9	4dsw4Ca4S	4dsw + 4[Mg^2+^ + SO_4_^2−^]
10	4dsw2Mg2S	4dsw + 2[Mg^2+^ + SO_4_^2−^]
11	4dsw4Mg4S	4dsw + 4[Mg^2+^ + SO_4_^2−^]

**Table 3:** Composition of brines.

Ions	FW	SW	1	2	3	4	5	6	7	8	9	10	11
	(ppm)	(ppm)	(ppm)	(ppm)	(ppm)	(ppm)	(ppm)	(ppm)	(ppm)	(ppm)	(ppm)	(ppm)	(ppm)
Na^+^ + K^+^	81,600	3240	810	810	810	810	810	810	810	810	810	810	810
Ca^2+^	1470	350	88	175	350	88	88	88	88	175	350	88	88
Mg^2+^	9540	740	185	185	185	370	740	185	185	185	185	370	740
Cl^−^	90,370	5440	1360	1360	1360	1360	1360	1360	1360	1360	1360	1360	1360
SO_4_^2−^	0	3010	753	753	753	753	753	1505	3010	1505	3010	1505	3010
HCO_3_^−^	0	220	55	55	55	55	55	55	55	55	55	55	55
TDS	182,980	9760	2440	2528	2703	2625	2995	3193	4698	3280	4960	3378	5253

**Table 4:** Stability indicator [56].

Zeta Potential Values (mV)	Stability
−25 and below	Highly stable
−20 to −25	Stable
−15 to −20	Less stable
−13 to −14	Fairly stable
−10 to −12	Unstable
−9 and above	Highly unstable

**Table 5:** Rate of change and acceleration of contact angle at different temperatures.

EW-NF (ppm)	Rate of Wettability Alteration (Deg./h)	
	Low Temp.	High Temp.
4dsw-NP	0.49	1.04
4dsw2Ca-NP	0.31	0.36
4dsw4Ca-NP	0.36	0.65
4dsw2Mg	0.33	1.19
4dsw4Mg-NP	0.30	1.32
4dsw2S-NP	0.07	1.05
4dsw4S-NP	0.48	1.58
4dsw2Ca2S-NP	0.56	1.42
4dsw4Ca4S-NP	0.16	1.48
4dsw2Mg2S-NP	0.33	1.67
4dsw4Mg4S-NP	0.25	1.53

## Nomenclature

4dsw	Four times dilution of Caspian Sea Water
BPR	Back Pressure Regulator
CA	Contact Angle
cc	cm^3^
CSW	Caspian Sea Water
DSW	Diluted Sea Water
EOR	Enhanced Oil recovery
EW	Engineered Water
EW-NF	Engineered Water Nanofluid
FW	Formation Water
IFT	Interfacial Tension
LSW	Low Salinity Water
LSWF	Low Salinity Water Flooding
MCR	Modular Compact Rheometer
NF(s)	Nanofluid (s)
NNF	Nanofluid Fluid Flooding
NP(s)	Nanoparticle(s)
OBR	Oil-brine-rock
OCA	Oscillating Contour Angle
OIIC	Oil initially in core
OOIC	Oil originally in core
PDI	Potential Determining Ions
PV	Pore Volume
RF	Recovery Factor
SC	Secluded Cavity
SDP	Structurally disjoining pressure
SEM	Scanning Electron Microscope
SW	Sea Water
TDS	Total Dissolved Solid
TFT	Thin-Film-Transistor
wt%	Weight %
